# Barriers and Facilitators to the Delivery of Physical Activity Promotion by Healthcare Professionals for Adults With Type 2 Diabetes: A Mixed‐Methods Systematic Review Using the Theoretical Domains Framework

**DOI:** 10.1155/jdr/4048417

**Published:** 2026-03-23

**Authors:** Emma Gibson, Angela Tufte-Hewett, Atiya Kamal

**Affiliations:** ^1^ Department of Psychology and Counselling, Birmingham City University, Birmingham, UK, bcu.ac.uk; ^2^ School of Health Sciences, University of Birmingham, Birmingham, UK, birmingham.ac.uk

**Keywords:** behaviour change, diabetes mellitus, healthcare professionals, mixed methods, physical activity, systematic review, Theoretical Domains Framework, Type 2

## Abstract

**Background:**

Physical activity is an integral component in the treatment and management of Type 2 diabetes. It is recommended that healthcare professionals (HCPs) promote physical activity to patients with Type 2 diabetes; however, they report many challenges to doing this in practice.

**Aims:**

This systematic review is aimed at identifying HCPs′ barriers and facilitators to promoting physical activity among adults with Type 2 diabetes.

**Methods:**

A mixed‐methods systematic review (MMSR) was conducted using a convergent integrated approach, following the Joanna Briggs Institute methodological procedures. MEDLINE, PubMed, PsycINFO, CINAHL, Web of Science and grey literature were searched from 1951 to 2020, with updates in 2024. Studies were excluded if they lacked HCPs′ views on Type 2 diabetes care or were not in English, but no study design restrictions were applied. Barriers and facilitators were coded to the Theoretical Domains Framework (TDF); each domain was ranked for importance, and an inductive thematic synthesis was conducted.

**Results:**

A total of 29 primary studies were included in the review. Barriers were reported across 11 TDF domains and facilitators across eight. Key findings within these TDF domains include insufficient time and resources to promote physical activity, a lack of organisational support, lack of knowledge and skills, challenges related to patient comorbidities/complications and HCPs′ perceptions of their roles and responsibilities in promoting physical activity.

**Conclusions:**

The findings highlight the need for change at HCP, organisational and environmental levels. Key improvements include clearer roles for promoting physical activity, system‐embedded prompts and the integration of disease‐specific modules into medical training.

**Statement**

This manuscript is based on work originally presented in the doctoral thesis of Emma Gibson, Exploring Healthcare Professionals′ Physical Activity Promotion for Adults With Type 2 Diabetes in Oman, Birmingham City University, 2025 [1].

## 1. Introduction

Type 2 diabetes is a complex and chronic disease that has reached epidemic proportions globally [2]. Although factors such as genetics, age, ethnicity, race and social determinants are known risk factors for Type 2 diabetes, lifestyle factors such as diet, physical activity and smoking can increase an individual′s susceptibility [3]. Regular physical activity of at least 150 min of moderate‐intensity activity or 75 min of vigorous‐intensity activity, combined with regular resistance training, is associated with improved health outcomes, including weight loss, reduced risk of cardiovascular disease and improved glycaemic control and lipid levels [4–6]. Despite the known benefits of physical activity, many people with Type 2 diabetes do not meet the recommended physical activity levels to achieve these improved outcomes [7, 8]. People living with Type 2 diabetes report multiple barriers to physical activity, such as lack of time, lack of knowledge, lack of infrastructure and facilities, social and cultural issues and inadequate emphasis on physical activity promotion by HCPs [9, 10].

Clinical practice guidelines recommend that HCPs promote physical activity to patients with Type 2 diabetes [4, 11, 12], but HCPs report that this is challenging in practice, resulting in a brief, nonspecific, inconsistent or missed advice [13–17]. Although a body of literature has examined factors influencing HCPs′ physical activity promotion, the evidence is conceptually and methodologically heterogeneous. Consequently, it remains unclear which determinants are most influential, how they interact and which should be prioritised when designing interventions to support HCPs [11, 18]. Together, these issues underscore the need for a systematic, theory‐informed synthesis to organise existing evidence within a coherent framework, clarify key determinants of HCPs′ physical activity promotion and inform the development of targeted, contextually relevant intervention strategies.

Evidence indicates that interventions explicitly informed by behavioural theory are more effective than those that are not, as theory helps to identify the key drivers of behaviour and supports adaptation to context [12–14]. Consequently, behaviour change theories and frameworks are recommended for developing interventions and implementation strategies, as they offer valuable insights into the individual, contextual, societal and environmental factors that influence behaviour and behaviour change [15–18]. The Theoretical Domains Framework (TDF) synthesises constructs from 33 theories of behaviour and behaviour change into 14 domains. The TDF is a recommended framework in implementation research, widely used to examine HCPs′ behaviours [15]. It offers a comprehensive and systematic approach to identifying barriers and facilitators to behaviour, thereby supporting efforts to bridge the evidence–practice gap [15].

The COM‐B model [19] is commonly used alongside the TDF as a complementary framework to identify influences on HCPs′ clinical practice behaviours [20–23]. Although the TDF enables detailed and systematic identification of behavioural determinants, the COM‐B model provides a higher level of conceptual structure for understanding how these determinants influence behaviour through the interaction of capability (psychological and physical), opportunity (social and physical) and motivation (automatic and reflective). Mapping TDF domains onto COM‐B enhances analytical rigour and comprehensiveness. Furthermore, it supports the translation of detailed findings into intervention design by aligning with the Behaviour Change Wheel [19] and facilitates the synthesis and prioritisation of determinants to inform resource allocation. This dual‐framework approach also enables clearer communication of findings to multidisciplinary implementation teams and policymakers within a coherent, theory‐based framework [19].

To better understand the barriers and facilitators HCPs face when promoting physical activity to patients with Type 2 diabetes, particularly given the variability in research settings and approaches, a comprehensive synthesis of existing evidence was needed. To achieve this, an MMSR in accordance with the Joanna Briggs guidelines [24, 25], was conducted to examine qualitative, quantitative and mixed‐methods studies. This MMSR aimed to synthesise, assess and develop an in‐depth understanding of HCPs′ experiences with promoting physical activity for patients with Type 2 diabetes.

## 2. Method

This systematic review followed the Joanna Briggs Institute (JBI) methodological procedures for MMSRs, [25] as well as the Preferred Reporting Items for Systematic Reviews and Meta‐Analyses (PRISMA) statement [26]. The protocol was registered in PROSPERO (CRD42020162844).

### 2.1. Inclusion Criteria

This review considered studies that included HCPs (aged 18+ years) working in any healthcare setting caring for adult patients with Type 2 diabetes. Intervention‐ and nonintervention‐based studies using quantitative, qualitative or mixed‐methods designs were also eligible for inclusion. Studies reporting perspectives on physical activity promotion from multiple people (e.g., patients and HCPs) and studies focusing on various components of Type 2 diabetes management were included if they addressed physical activity; however, only data relating to HCPs and physical activity were included in the review. Studies that reported only on the effectiveness of physical activity interventions were excluded, as were studies that did not report HCP perspectives on promoting physical activity among adults with Type 2 diabetes or that reported only patient perspectives. Studies that did not include a physical activity element were not written in English, and protocols and conference reports were also excluded. See Table 1 for an overview of the eligibility criteria in accordance with the elements from ‘PIO’ (Population, Intervention, Outcome).

**Table 1:** Search terms based on the PIO framework for the MEDLINE and PubMed searches.

PIO criteria	Search term
Patient/population/problem	Health personnel∗ OR health occupations∗ AND diabetes mellitus OR Type 2 OR Type 2 diabetes∗ or Type 2 diabetes mellitus∗ OR t2dm∗
Intervention/issue	Exercise∗ OR Sports∗OR sedentary behavio#r∗ OR exercise therapy∗ OR physical fitness∗ OR lifestyle∗
Outcome	Intervention∗ OR early medical intervention∗ OR internet‐based intervention∗ OR health promotion∗ OR patient care management∗ OR counsel#ing∗ or counsel∗ OR program evaluation∗ OR health education∗ OR delivery of health care∗

### 2.2. Search Strategy

A search strategy was developed to identify published and unpublished studies and to determine the search terms for the research questions, which were mapped onto relevant eligibility criteria from ‘PIO’. The search strategy was developed collaboratively with an academic librarian and by consulting the published literature to ensure comprehensive coverage. A combination of MeSH (Medical Subject Headings) keywords and Boolean operators was used. The full search strategy is provided in the supporting materials (See Appendix S1).

Searches were conducted in MEDLINE, PubMed, CINAHL, PsycINFO and Web of Science. Grey literature sites (Ethos, Open Grey and Google Scholar) were also searched, as were the reference lists of the included articles. Grey literature was searched to minimise publication bias; however, no papers were found. All searches were limited to English‐language human studies from 1951 to 2024. The same searches were conducted again in September 2025, but no new articles were retrieved.

### 2.3. Study Selection

All search results were imported into EndNote X7, and duplicates were removed. The remaining articles were imported into Covidence, an online systematic review management tool, where titles and abstracts were independently screened by two reviewers (E.G. and A.T‐H.) against the inclusion criteria. Studies not excluded at this stage were retrieved for full‐text screening, which was independently undertaken by two reviewers (E.G. and A.K.). Full‐text articles that did not meet the inclusion criteria were excluded, with the reasons documented.

Interrater reliability was calculated using Cohen′s kappa, which measures agreement beyond chance on a −1 to 1 scale [27]. For this MMSR, agreement was fair at title/abstract screening (*κ* = 0.29) and moderate at full‐text screening (*κ* = 0.50). Interrater reliability reflects the consistency of reviewer decisions and underpins the validity of systematic reviews [28]. Higher kappa values (≥ 0.61) indicate strong agreement, whereas lower values (≤ 0.40) suggest greater variability in judgement [29]. Discrepancies at either screening stage were resolved through discussion between the two reviewers. Where consensus could not be reached, a third reviewer (A.T‐H. or A.K.) was consulted. When further information was required to assess eligibility or only a study protocol was available, the corresponding author was contacted via email to request the necessary details.

### 2.4. Assessment of Methodological Quality

An assessment of the methodological quality of all full‐text studies included in the review was conducted by E.G. Quantitative studies, quantitative and qualitative components of mixed‐method studies and qualitative studies were appraised using the standardised JBI critical appraisal tools [30, 31]. For quantitative studies, the checklist for analytical cross‐sectional studies was used. For qualitative studies, the checklist for qualitative research was used. In accordance with JBI guidance, the mixed‐methods study was assessed using the cross‐sectional and qualitative quality assessment tools. To assess the quality of the included studies, each study was reviewed and scored according to the relevant JBI criteria. Studies were given a score of 1 when the JBI criterion was met, or 0 if it was not met or unclear. For qualitative studies, the total score is 10; for quantitative studies, the total possible score is 8. For each study, a summary quality score was computed based on the percentage of checklist criteria that were fulfilled. Studies were classified as low, moderate or high quality based on the ranges of summary scores: 0%–39%, 40%–69% and ≥ 70%, respectively [32]. A second review author (A.T‐H.) independently assessed all included studies. There were minor differences in study quality ratings, which were resolved with discussion. All studies were included in the MMSR regardless of methodological quality.

### 2.5. Data Extraction and Analysis Plan

Data from each of the included studies were extracted deductively using framework synthesis [33, 34] and directed content analysis [35] using the TDF [15] as an a priori framework. This approach provides a systematic method for integrating established frameworks into the research process, enabling a focused yet comprehensive examination of a topic [33]. Framework synthesis utilises a five‐step deductive and inductive approach to code and analyse extracted data, which consists of (1) familiarisation, (2) identification of the thematic framework, (3) indexing, (4) charting and (5) mapping and interpretation.

#### 2.5.1. Stage One: Familiarisation

This was an iterative process that involved reading and rereading the included studies to ensure a comprehensive understanding of the content and to identify all barriers and facilitators.

#### 2.5.2. Stage Two: Framework Selection

The TDF [15] informed the development of an a priori framework to extract data whereby, for coding purposes, the 14 domains of the TDF represented the parent node and the barriers or facilitators the child nodes.

#### 2.5.3. Stage Three: Indexing

The contextual details of each study were captured using an extraction form developed for this review. Data were extracted from both qualitative and quantitative studies, and where necessary, in accordance with the JBI convergent integrated approach to MMSRs; numerical data were qualitized (transformed from numerical to textual description or narrative interpretations) [25]. The extracted data, including those that were qualitized, were imported into Atlas.ti and mapped to the TDF domain and the corresponding COM‐B component, they best represent. At this point, all three reviewers met to verify and discuss 20% of the coded data to ensure agreement with the coding process. Both the qualitative data and the qualitized quantitative findings were coded deductively to the TDF and COM‐B model. During indexing, each extracted meaning unit was examined and assigned to the TDF domain and corresponding COM‐B component that best captured its behavioural determinant. Inductive coding was then conducted to identify additional concepts not accounted for by the framework, ensuring that no relevant themes were overlooked.

#### 2.5.4. Stage Four: Charting

Similar data within TDF domains were synthesised, and themes and subthemes were developed in accordance with thematic synthesis guidance [36]. There are three key components to thematic synthesis; the first stage, line‐by‐line coding of the data, was completed at stage three of the framework synthesis described above. Component two involves generating descriptive themes that remain close to the data, and component three involves generating analytical themes that involve the reviewer′s interpretation of the descriptive themes, which were conducted at the charting stage of framework synthesis. Literature related to the TDF [15] and framework synthesis [33] suggest that the selected a priori framework may not sufficiently capture all the extracted data, and, as such, there is a risk of missing important influencing factors not considered by the selected framework. To mitigate this, as is recommended in the literature, an inductive analysis was conducted after the deductive analysis. At this fourth stage, the findings from Stage three (indexing) were analysed using inductive thematic synthesis [36].

#### 2.5.5. Stage Five: Mapping and Interpretation

The analytical themes were written up and presented in narrative form. In addition, the TDF domains were ranked for importance using three established criteria: (1) frequency (the number of included studies contributing data to each domain), (2) elaboration (the number of distinct themes and subthemes identified within a domain) and (3) presence of conflicting beliefs within or across studies [15, 37, 38]. After coding all data to the TDF, each domain was assessed against these criteria, and domains with higher frequency, greater elaboration and evidence of conflicting beliefs were considered more influential. This ranking process provided a comprehensive understanding of the barriers and facilitators to physical activity promotion across the TDF domains and insight into which domains most influenced HCPs′ physical activity promotion. A deductive content analysis approach [39] was applied to structure this process, allowing the systematic comparison of domains and their relative importance.

## 3. Results

### 3.1. Search Results

**Figure 1:**
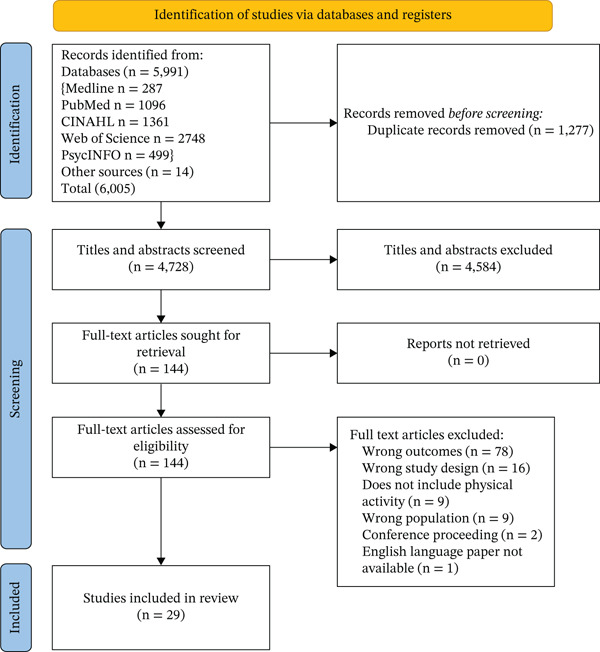
PRISMA of studies included and excluded from the review of barriers and facilitators. Studies included were *n* = 29 from database searches.

**Figure 2:**
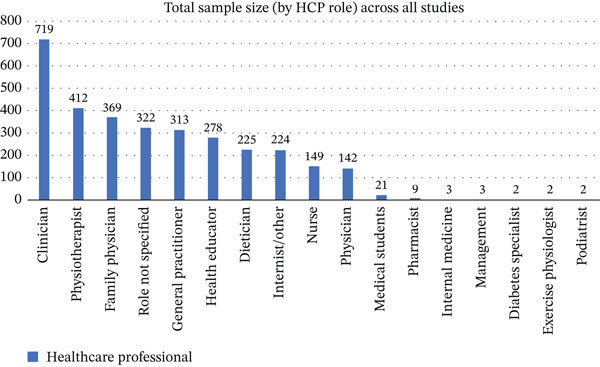
Total HCPs (by role) included across all studies.

A total of 6005 citations were retrieved from the database searches. After removing 1277 duplicates, the titles and abstracts of 4728 studies were screened, of which 144 full‐text articles were retrieved and reviewed. One hundred fifteen articles were excluded, and 29 articles published between 1993 and 2024 fulfilled the inclusion criteria and were included in the review (see Figure 1). Of the 29 articles included in the review, 15 were qualitative studies [40–54], 13 were quantitative studies [55–65] and one was a mixed‐methods study [66]. Table 2 provides an overview of the characteristics of the included studies, and Figure 2 presents the distribution of healthcare professionals in the sample across all included studies.

**Table 2:** Characteristics of the included studies.

Author, date	Country/setting	Research aim	Study characteristics	Participant characteristics	Findings
Abouammoh et al. (2016) [40]	Saudi ArabiaHospital and primary healthcare	To explore the experiences of GP international medical graduates (IMGs) providing lifestyle advice to people with Type 2 diabetes and to raise awareness about cross–cultural interaction	QualitativeFocus groups and one‐to‐one interviews purposive samplingThe analytic technique used was not stated.	Total *n* = 47IMGs from the hospital setting *n* = 21GP IMGs working in primary care *n* = 15IMG that took part in follow‐up interviews *n* = 11	HCPs were aware of local cultural norms and believed they were providing culturally appropriate advice to patients. Yet, they were aware that patients felt that their advice was not always culturally relevant, and this affected their confidence in HCPs′ ability to provide guidance in this area of care
Alghafri et al. (2017) [41]	OmanPrimary health care	To determine the perceptions of HCPs on physical activity promotion for adults with Type 2 diabetes within a local clinical primary care setting in Oman	QualitativeFocus groupsPurposive samplingThematic analysis	Total *n* = 29Family physicians *n* = 17Nurses *n* = 5Health educators *n* = 3Dieticians *n* = 4	Key barriers identified were related to the healthcare system (e.g., lack of physical activity guidelines), individual factors (e.g., social norms that discourage activity) and environmental limitations (e.g., insufficient facilities). Recommendations included assigning the role of physical activity promotion to dietitians and using technology (e.g. pedometers) to support patients
Armstrong‐Shultz et al. (2001) [55]	United StatesNot specified	To compare and contrast patient and practitioner barriers to following meal or exercise plans and identify patient characteristics related to their perceived barriers	QuantitativeCross‐sectional survey, questionnaire—developed by the researchersPurposive samplingDescriptive statistics, ordinal logistic regression, principal components factor analysis.	Total *n* = 143Diabetes educators *n* = 143	HCPs′ perceptions of patient barriers that would stop them from promoting physical activity (% of HCPs):A lack of time (52%), physical limitations (49%), physical pain (48%), balancing exercise with food intake (44%), low priority given to exercise (41%), dependence on weather conditions (37%), lack of exercise space (35%), physical discomfort (34%), inconvenient location for exercise (32%), lack of necessary equipment (28%), a dislike of sweating (20%), concerns about being overweight (13%) and concerns about low blood sugar (11%).
Avery (2014) [42]	United KingdomPrimary care	To explore the experiences of HCPs in promoting physical activity and understand their training needs	QualitativeSemistructured interviewsConvenience samplingContent analysis	Total *n* = 5GP *n* = 3Specialists in diabetes/practice lead for diabetes *n* = 2	Barriers included difficulties communicating complex medical information to patients, low patient engagement and reluctance to take responsibility for managing their condition, a lack of training, a lack of tools to assess patients and that prescribing medication was more straightforward than physical activity promotion
Berry et al. (2012) [43]	CanadaUnclear	To examine diabetes educators′ understanding of how clients understand and use knowledge regarding nutrition and physical activity, the barriers diabetes educators face when translating lifestyle guidelines and what factors the educators believe influence the uptake of guidelines by those with Type 2 diabetes	QualitativeSemistructured interviewsTypical case samplingCross‐case analyses at the individual educator level	Total *n* = 13Registered nurses *n* = 2Registered dietitians *n* = 7Manager in charge of developing and reviewing educational materials *n* = 1Role not specified *n* = 3	Influences on HCPs physical activity promotion included the complexity of psychological, cultural and social influences on patients′ abilities to adopt new behaviours, limited resources and a lack of support from exercise specialists and other HCPs
Carbone et al. (2007) [44]	United StatesHealth centre	To examine HCPs perceptions of patient barriers and facilitators to adopting self‐ management strategies and experiences supporting patients′ self‐management strategies	QualitativeFocus groupsPurposive samplingContent analysis/topical analysis	Total *n* = 8Medical paraprofessionals, nurses, educators, outreach workers, physicians, physician assistants and nurse practitioners.	Barriers reported by HCPs included patients attitudes towards physical activity, limited safe spaces for physical activity, limited access to exercise facilities integrating exercise into the patients′ daily routines and difficulty in effectively communicating the benefits of regular physical activity
Dillman et al. (2010) [56]	CanadaUnclear	To examine diabetes educators′ perceptions of their abilities, attitudes and difficulties related to physical activity and exercise counselling. To explore diabetes educators′ perceptions of their patients′ abilities and attitudes related to physical activity and exercise in managing their diabetes. Finally, examine the self‐reported barriers diabetes educators face concerning physical activity and exercise counselling	QuantitativeA cross‐sectional survey, questionnaire developed by the researchersPurposive samplingDescriptive, frequency analysis and MANOVA	Total *n* = 119Diabetes educators *n* = 119	Barriers reported as % (*n*), mean frequency (1–4 scale), mean impact on counselling (1–4 scale)Lack of time to counsel 65 (77), 3.22, 2.70Lack of interest by Patient 34 (40), 3.00, 2.92Lack of resources −30 (36), 3.45, 3.05HCP lack of ability/knowledge −29 (34) =, 3.05, 2.69Low counselling and referral efficacy (Wilk^′^s lambda = 0.84, *p* = 0.003).
Doehring et al. (2016) [67]	CanadaPrimary, secondary, tertiary and community care	To examine the knowledge, attitudes and practices of physiotherapists in preventing and managing diabetes	QuantitativeA cross‐sectional survey, questionnaire Convenience samplingDescriptive statistics using Microsoft Excel and percentages calculated	Total *n* = 401Physiotherapists *n* = 401	Fewer than half (46.6%) of the respondents knew the recommended amount of weekly aerobic activity (150 min).16.7% of physiotherapists were aware of the Canadian Diabetes Association Clinical Practice Guidelines76.3% of participants reported that their education had not adequately prepared them to effectively manage people with diabetes
Dranebois et al. (2019) [57]	French GuianaPrimary care	To evaluate the barriers, facilitators and practices of GPs regarding the prescription of physical activity for patients with Type 2 diabetes	QuantitativeA cross‐sectional survey, questionnaire purposive samplingDescriptive statistics, chi‐squared independence tests and Fisher′s exact test	Total *n* = 73Physicians *n* = 73	Barriers identified by participants included lack of structure (91.8%), lack of training and knowledge (80.8%), unsuitable support (75.3%), lack of support (74%), refusal of the patient (71.2%), lack of time (69.9%), language barrier (58.9%), no dedicated pricing (54.8%) and not a reason for consultation (53.4%)
George et al. (2006) [58]	United StatesInpatient and outpatient settings	To determine if differences existed between noncertified diabetes educator registered dietitians (non‐CDE‐RDs) and CDE‐RDs certified diabetes educator registered dietitians (CDE‐RDs)	QuantitativeA cross‐sectional survey, questionnaire purposive samplingDescriptive statistics, frequencies, independent samples *t*‐test and two‐way ANOVA and post hoc tests on significant findings	Total *n* = 167Non‐CDE RDs *n* = 73CDE RDs *n* = 94	Knowledge scores: CDE‐RDs 11.8 versus non‐CDE‐RDs 11.1Design scores: CDE‐RDs 33.5 versus non‐CDE‐RDs 29.2Content scores: CDE‐RDs 26.9 versus non‐CDE‐RDs 22.4Total exercise teaching scores: CDE‐RDs 60.4 versus non‐CDE‐RDs 51.6
Gross et al. (2007) [59]	IsraelCommunity‐based primary care	To examine primary care physicians′ self‐ efficacy in counselling diabetic patients on lifestyle behaviours and the association between self‐efficacy and self‐reported counselling practices for these patients	QuantitativeA cross‐sectional survey, questionnaire purposive samplingChi‐squared tests and logistic regression	Total *n* = 743GP *n* = 238Family physician *n* = 342Internist/other *n* = 163	Self‐efficacy on exercise counselling: 27% of physicians felt they could very effectively influence their patients to exerciseSelf‐efficacy impact on exercise counselling: Physicians with high self‐efficacy discussed exercise with almost all their patients 85.4% of the time, compared with 72.4% for those with low self‐efficacy
Hixenbaugh and Winkley (2001) [60]	United KingdomNot specified	To compare patient and HCPs perceptions of the aspects of diabetes self‐management most frequently omitted by patients	QuantitativeA cross‐sectional survey, questionnaire purposive samplingBetween‐group analyses X^2^‐ test.	Total *n* = 36	91.4% of HCPs believed that patients were likely to omit regular exercise from diabetes management.85.7% of HCPs considered patients′ emotional stress as a barrier.74.3% of HCPs reported lifestyle interference as a barrier to physical activity, resulting in pessimism from them about promoting physical activity.48.6% identified patients′ lack of time as a barrier.
Hnatiuk et al. (2012) [61]	CanadaPrimary care	To examine perceptions of physical activity support provided by physicians, nurses and other HCPs to adults with Type 2 diabetes and compare these perceptions with those of the patients	QuantitativeThe researchers developed a cross‐sectional survey questionnaire, the physical activity support questionnairePurposive sampling Kruskal–Wallis	Total *n* = 48Nurses *n* = 18Physician *n* = 15Other HCPs *n* = 15	17% of healthcare providers (HCPs) could identify the Canadian Diabetes Association (CDA) guidelines for physical activity without prompting.12% of HCPs identified Canada′s Physical Activity Guide (CPAG) unprompted.
Jones et al. (2014) [45]	AustraliaNot specified	To identify barriers and facilitators to effective Type 2 diabetes self‐management in rural settings with patients and HCPs	QualitativeSemistructured telephone interviews purposive samplingInductive thematic analysis	Total *n* = 18Diabetes educators *n* = 10Podiatrists *n* = 2Nurses *n* = 3Dieticians *n* = 3	Barriers include patients′ time management, motivation, denial of illness, access to resources, community attitudes and patient and HCPs education gaps. Supportive relationships were reported as facilitators.
Karduck and Chapman‐Novkofski (2018) [62]	United StatesNot specified	To develop and administer a questionnaire examining factors associated with health app use, recommendations and effectiveness	QuantitativeThe researchers developed a cross‐sectional survey questionnaire—The Clinician Apps SurveyPurposive samplingDescriptive statistics, chi‐square test of independence	Total *n* = 719Clinicians *n* = 719	58% of clinicians recommended apps to track physical activity62% of clinicians used apps for assessing physical activity among their patients58% believed apps were superior to traditional methods for tracking physical activity
Khairnar et al. (2018) [68]	United StatesPrimary care	To examine HCPs perspectives on barriers and facilitators to Type 2 diabetes self‐management	QuantitativeA cross‐sectional survey, questionnaire—developed by the researchersPurposive samplingDescriptive statistics, frequencies and percentages	Total *n* = 24Physicians *n* = 21Physician assistant *n* = 1Transition of‐care liaison *n* = 1Nurse practitioner *n* = 1	95.2% of HCPs considered regular moderate exercise extremely important for diabetes self‐management85.71% of HCPs perceived regular moderate exercise as at least difficult for their patients76.19% of HCPs believed that less than 50% of their patients were adherent to regular moderate exercise
Lanhers et al. (2015) [63]	FranceNot stated	To examine if there was a link between physical activity in Type 2 diabetic patients and GPs′ attitudes to physical activity promotion	QuantitativeA cross‐sectional survey, questionnaire cluster samplingDescriptive statistics, frequencies, percentages, Fischer′s exact test, Spearman′s correlation coefficient test, Wilcoxon test, one‐way Kruskal–Wallis ANOVA, random effect models	Total *n* = 48GPs *n* = 48	A significant correlation where higher barrier scores among GPs are associated with higher barrier scores among their Type 2 diabetes patients (*p* = 0.03)A high intraclass correlation of 34% indicated a significant influence of GPs′ attitudes towards prescribing physical activity on their patients′ activity levels
Larme and Pugh (1998) [66]	United StatesPrimary care	To examine attitudes of primary care providers towards diabetes care	Mixed‐methodsA cross‐sectional survey, questionnaire One to one semi‐structured interviews purposive samplingNonparametric quantile testContent analysis	Total *n* = 31Physicians *n* = 24Mid‐level providers (family nurse practitioners and physician assistants) *n* = 7	Diabetes was considered significantly more challenging to treat compared to hypertension, with 24 out of 30 respondents rating it above 5.5 (*p* < 0.001), and angina, with 20 out of 30 rating it above 5.5 (*p* = 0.03). Additionally, the majority rated hyperlipidaemia (18 out of 30) and arthritis (18 out of 30) as easier to manage than diabetes.The participants reported a lack of training to support patients in changing their physical activity behaviour, which was exacerbated by patients ingrained habits and the complexities of treatment
Matthews et al. (2014) [46]	United KingdomPrimary and secondary care	To explore the views of HCPs on physical activity promotion in routine diabetes care	QualitativeSemistructured interviews and online survey purposive samplingInterpretative phenomenological analysis	Total *n* = 16 for the online survey (open‐ended questions)Two management *n* = 2Three consultant physicians *n* = 3Six diabetes nurse/practice nurses *n* = 6 GPs *n* = 4Anonymous *n* = 1Total *n* = 7 for interviewsParticipants were from primary care, secondary care and health service management.	Key findings included a lack of structure for physical activity promotion, insufficient resources, lack of role clarity, inadequate behaviour change training, lack of awareness of a pilot referral programme and difficulties prioritising physical activity in routine appointments
Miller and Beech (2009) [47]	United StatesRural community centres	To evaluate rural HCPs physical activity counselling experiences and their perceptions of motivational interviewing (prior to training)	Qualitative Focus groupsPurposive samplingContent‐based analysis and thematic coding	Total *n* = 33Nurses dieticiansCertified diabetes educators physician	Participants reported low comfort with physical activity counselling due to a lack of knowledge or feeling hypocritical about their own inactivity. Those who were regularly active and had counselling experience felt more comfortable. Although motivational interviewing was seen as promising, time constraints and limited provider input were noted as barriers
Mogre et al. (2019) [48]	GhanaTertiary care	To explore HCPs and patient perspectives on barriers to self‐management for Type 2 diabetes	QualitativeSemistructured interviewsPurposive sampling and snowballing techniques constant comparative method	Total *n* = 14Nurses *n* = 8Physician assistants or prescribers *n* = 2Nutrition officers *n* = 2Dieticians *n* = 2	HCPs’ perceptions of patients were barriers to physical activity promotion, including patients′ lack of motivation or willingness to exercise, beliefs that diabetes is caused by spiritual forces, inadequate family support, social stigma, low‐income levels, limited access to exercise facilities, busy work schedules and long distances to hospitals
Paiva et al. (2019) [49]	PortugalPrimary and tertiary care	To explore patient and provider perceptions of barriers and facilitators to patient‐centred communication for Type 2 diabetes	Qualitative focus groups purposive sampleGrounded theory	Total *n* = 33Primary care physicians, nurses, nutritionists, pharmacists, ophthalmologists, vascular surgeons, psychologists, nephrologists, endocrinologists	Patients complications were barriers to HCPs providing physical activity advice. Increasing patients access to resources to support behaviour change were facilitators
Powell et al. (2016) [64]	United StatesPrimary care, secondary care and tertiary care	To examine factors influencing physical activity counselling by diabetes educators delivering diabetes self‐management/support	QuantitativeCross‐sectional survey study, questionnaire purposive samplingDescriptive statistics, Kruskal–Wallis *H* test, Mann–Whitney *U* test, post hoc analysis	Total *n* = 119Nurses *n* = 72Nutritionist *n* = 34Pharmacist *n* = 7Health educators *n* = 3Physician *n* = 2Exercise physiologist *n* = 1	Diabetes educators spent approximately 14.5 minon physical activity, less than on diet and medication management74% know the moderate‐intensity aerobic activity guideline, 20.5% for vigorous intensity and 62.8% for resistance training54.7% of educators are very confident in their physical activity counsellingKey challenges include patient engagement and limited time during sessions
Raaijmakers et al. (2013) [50]	The NetherlandsPrimary care Secondary care	To explore HCPs perceived barriers to diabetes care	QualitativeOne‐to‐one semistructured interviews random samplingMethod of analysis not stated	Total *n* = 18Family physicians *n* = 3Practice nurses *n* = 3Diabetes nurses *n* = 2Dieticians *n* = 3Physical therapists *n* = 2Internal medicine *n* = 3Pharmacists *n* = 2	Lack of role clarity, lack of knowledge and awareness of local community resources and lifestyle programmes and collaborative opportunities with physical activity experts were reported as barriers. Compiling list of local physical activity resources for patients was a facilitator
Ruby et al. (1993) [65]	United StatesInpatient and outpatient	To examine registered nurses, diabetes educators exercise teaching programmes for elderly patients with non‐insulin‐dependent diabetes mellitus (NIDDM)	QuantitativeA cross‐sectional survey, questionnaire random samplingDescriptive statistics, student *t*‐tests, Mann–Whitney *U* tests, Spearman′s Rho and Pearson′s *r*	Total *n* = 197	46% of educators cited lack of resources as a primary barrier.30% noted a lack of specific knowledge on exercise prescription.29% mentioned negative stereotypes, like ageism, affecting exercise teaching for elderly clientsFacilitators included experience and education. RN, CDEs with over 30 weekly work hours and regular continuing education had more comprehensive programmes
Stuij (2018) [51]	The NetherlandsPrimary and secondary care	To provide in‐depth insight into the experiences of Dutch HCPs delivering physical activity counselling to Type 2 diabetic adults	QualitativeOne‐to‐one interviewsPurposive samplingNarrative approach	Total *n* = 24Physiotherapists *n* = 8Practice nurses *n* = 5Diabetes nurses *n* = 3GPs *n* = 2Internist *n* = 1Diabetes specialist nurse *n* = 1Dietician *n* = 1Exercise coach *n* = 1Exercise expert *n* = 1Health specialist *n* = 1	Barriers included difficulty understanding patients′ perspectives, socioeconomic and cultural differences, patients′ lack of motivation, role clarity, lack of time, knowledge and training gaps. Facilitators included goal setting, personal experience with physical activity, motivational interviewing and initiating walking programmes.
Svenningsson et al. (2011) [52]	SwedenPrimary health care and county medical care settings	To gain a deeper understanding of HCPs′ main issues in consultations with diabetic and obese patients, and how these issues could be overcome	QualitativeGroup and individual interviewsOpen sampling and theoretical sampling grounded theory	Total *n* = 20Nurses *n* = 13Physicians *n* = 4Dieticians *n* = 2Physiotherapist *n* = 1	Barriers included time constraints, knowledge gaps, a focus on medical goals, resistance from patients and emotional burden. Facilitators included coaching and supportive approaches, individualised care strategies and collaboration with patients
Torres et al. (2010) [53]	BrazilPrimary care	To explore the perception of HCPs on their role in patient education for people with Type 2 diabetes	Qualitative–Case study Focus groupsPurposive samplingThematic analysis	Total *n* = 23	Barriers included perceptions of patients lack of time to adhere to healthy life habits, lack of money, absence of appropriate places for physical activity and individual passivity towards treatment
Zimmermann et al. (2018) [54]	AustraliaNot stated	To explore the consultation practices of accredited exercise physiologists with people with Type 2 diabetes and the recommendations they provide to promote long‐term adherence to physical activity	QualitativeFocus groups and semistructured interviews purposive samplingThematic analysis	Total *n* = 21	Barriers included a focus on medical information, a lack of psychosocial assessment tools, limited experience with behaviour change techniques, time constraints and client resistance. Facilitators included experience and rapport building, the use of motivational interviewing, adopting a person‐centred approach and group exercise sessions

### 3.2. Quality Appraisal of the Included Studies

A total of 14 qualitative studies were appraised to be of high quality [40–54], whereas one qualitative study [47] and the qualitative component of the mixed‐methods study [66] were appraised as moderate quality. Scoring for all studies is provided in the supporting materials (see Appendix S2).

Across all of the qualitative studies, congruence, defined by the JBI as alignment between a study′s philosophical perspective, methodology, research question or objectives, data collection methods, data analysis and interpretation of findings, was generally well‐demonstrated. All but one study demonstrated congruity between the research methodology and the research question or objectives, and with the exception of two studies [47, 66], methodology and data analysis were also evident.

All studies, except one [66], met the criteria for adequate participant representation and reported ethical approval of an appropriate body. Researcher influence, defined by JBI as acknowledgement of the reciprocal relationship between the researcher and the research, including reflection on how the researcher may have influenced the study and how the research context may have shaped the researcher, was addressed in seven studies. Across all studies, it was unclear whether congruence between the philosophical perspective and the methodological approach had been adequately considered, and none of the studies located the researcher culturally or theoretically.

Three of the quantitative studies were appraised as high quality [55, 58, 59], 10 were of moderate quality [56, 57, 60–65, 67, 68], and the quantitative component of the mixed‐methods study was appraised as low quality [66]. All studies used an appropriate statistical analysis. The inclusion criteria were clearly defined in most studies, except in four [56, 66–68]. Except for one study [63], all studies described the setting and participants in detail. Seven studies demonstrated the use of objective, standard criteria for measuring the condition (physical activity promotion for patients with Type 2 diabetes) [55–60, 67]. Confounding factors were identified in two studies; [59, 67] however, strategies to deal with these were not identified. Outcomes were measured validly and reliably in four studies [55, 58, 60, 68], but were unclear in the remaining studies.

### 3.3. TDF Domains

Barriers and facilitators were identified across all TDF domains except goals, intentions and reinforcement (see Table 3). Based on the importance criteria of frequency, elaboration and conflicting beliefs [69], the environmental context and resources domain was the highest‐ranked domain for influencing HCPs′ physical activity promotion. The environmental context and resources domain was also the highest‐ranked domain in terms of barriers, followed by beliefs about consequences, knowledge, skills, beliefs about capabilities and social/professional role and identity. The top five ranking domains represented 85% of barriers coded. For facilitators only, the goals domain ranked the highest, followed by environmental context and resources, social/professional role and identity, knowledge, beliefs about consequences, social influences, skills and behavioural regulation. See supporting materials Appendix S3 for an overview of the themes and subthemes, number of studies and whether they were identified as a barrier, facilitator or both.

**Table 3:** Ranking of TDF domain (with COM‐B) importance according to frequency, elaboration and conflicting beliefs for barriers and facilitators.

Ranking	TDF domain (COM‐B)	Frequency (no. of studies identified max *n* = 29)	Elaboration (number of themes/subthemes)	Evidence of conflicting beliefs (yes/no)
First	Environmental context and resources (physical opportunity)	21	8	Yes
Second	Beliefs about consequences (reflective motivation)	18	5	Yes
Third	Knowledge (psychological capability)	16	6	Yes
Fourth	Social/professional role and identity (reflective motivation)	12	3	Yes
Fifth	Skills (physical capability)	10	2	Yes
Sixth	Social influence (social opportunity)	8	1	Yes
Seventh	Beliefs about capabilities (reflective motivation)	8	1	No
Eighth	Goals (reflective motivation)	7	1	No
Ninth	Emotion (automatic motivation)	5	1	No
Tenth	Memory, attention and decision processes (psychological capability)	4	1	No
Eleventh	Optimism (reflective motivation)	3	1	No
Twelfth	Behavioural regulation (psychological capability)	2	1	Yes
No ranking	Intentions (reflective motivation)	0	0	—
No ranking	Reinforcement (automatic motivation)	0	0	—

### 3.4. Thematic Synthesis

The five highest‐ranking domains from the thematic synthesis are discussed in detail below. See the supporting materials, Appendix S4, for a detailed overview of the findings for each TDF domain and exemplar quotes.

#### 3.4.1. Environmental Context and Resources (Physical Opportunity)

A lack of organisational support was a barrier to physical activity promotion in seven studies [41, 42, 46, 50, 57, 64, 65]. These include a lack of training opportunities for physical activity promotion [42, 65], inadequate multidisciplinary collaboration [50], physical activity not being set as a quality indicator or an agreed healthcare delivery priority within the health system and insufficient support from other sectors or stakeholders [41, 46]. Financial challenges were reported in eight studies [41, 46, 51, 53, 57, 62, 64, 65]. These included a lack of financial reimbursement to HCPs for physical activity counselling or prescription [57, 64], short duration of insurance coverage for physiotherapy appointments for patients [51] and limited funding provided to develop adequate physical activity services or programmes to support HCPs in this area of their practice [46, 65].

Time‐related barriers were reported in 12 studies and included short hours of diabetes clinics [41], insufficient time to cover all aspects of diabetes treatment, including physical activity [46, 51, 54, 56, 57, 64, 65, 68], low frequency of appointments [51], HCPs work schedules [63], the large amount of data collection required during patients′ appointments [46, 54] and the need to focus on patients diabetes‐related complications or other chronic illnesses [68]. HCPs′ knowledge about patients′ lack of time for physical activity was also a barrier to promoting physical activity [41, 43–45, 48, 55]. When HCPs perceived physical activity was not a priority for patients due to their schedules or competing demands, such as social or work commitments, they found it more challenging to promote physical activity as they felt patients would be less receptive to their advice [41, 43, 45, 48, 53, 55].

Barriers related to access and availability of resources included inadequate staffing and support, unavailability of exercise specialists [41, 46, 65], inadequate physical activity guidelines [41, 43, 46, 57, 58, 61, 64], limited educational materials for patients and HCPs [41, 43, 46], inadequate physical activity referral pathways [41, 46, 50, 51, 57] and a lack of space for physical activity consultations within healthcare facilities [41]. HCPs reported that patients′ limited access to physical activity resources made it more challenging to offer realistic guidance and support [41, 45, 53, 55] and develop and implement effective exercise teaching programmes [65]. Barriers include facilities being too far away or closed, an unsupportive‐built environment, such as a lack of accessible and safe areas to walk [41], the patients′ rural location [45] and a lack of access to appropriate materials or equipment [55].

Facilitators to overcome resource‐related barriers included the development of a more conducive‐built environment to support patients to be more physically active, such as building more walking trails [49], compiling lists of local physical activity initiatives, opportunities and resources to recommend to patients [41, 50] and creating walking groups [51]. In one study, a pilot physical activity referral scheme was viewed as a facilitator of physical activity promotion; however, HCPs′ awareness of this scheme was low and was identified as a key training priority [46].

#### 3.4.2. Knowledge (Psychological Capability)

A lack of general knowledge about physical activity was identified by HCPs in 13 studies as a barrier to promoting physical activity [41–43, 46, 47, 56–58, 61, 62, 64, 65, 67]. Specific barriers included a lack of knowledge of basic information about physical activity and Type 2 diabetes, such as what safe blood glucose levels are during exercise [67], what physical activity (type, duration and frequency) to recommend to people with Type 2 diabetes [42], how physical activity affects diabetes control [64] and a lack of knowledge on how to develop appropriate physical activity plans for patients with complications or comorbidities, such as obesity, cardiovascular disease and osteoarthritis [41, 43, 46, 64, 65]. Limited knowledge, understanding and awareness of the published physical activity guidelines and policies were barriers to physical activity promotion in eight studies [41, 43, 46, 57, 58, 61, 64, 67].

Inadequate training in physical activity also had a detrimental impact on HCPs knowledge [41, 46, 56, 57, 66]. HCPs reported that their education and training in medical schools or residencies were insufficient to equip them with the knowledge to promote physical activity effectively [40, 67]. Knowledge of patients′ social and environmental context facilitated physical activity promotion in studies conducted in Oman [41] and Saudi Arabia [40]. HCPs reported that, despite challenges such as hot climates, inadequate infrastructure, rapid urbanisation and gender norms that can hinder patients′ uptake of physical activity, strategies were used to promote physical activity. These included encouraging male patients to walk to more distant mosques for prayer, finding ways for females to be physically active at home [40] and creating maps of exercise facilities in different areas, which allowed HCPs to provide specific recommendations on where and how patients could engage in physical activity [41].

#### 3.4.3. Beliefs About Consequences (Reflective Motivation)

Patient factors were also barriers to HCPs′ efforts to promote physical activity. In 12 studies, it was perceived that some patients were not interested or receptive to physical activity advice, that physical activity was not a priority, or that patients lacked motivation to change their behaviour [41–43, 46, 48, 50, 51, 54–57, 63]. Patient noncompliance with physical activity recommendations or unwillingness to change their behaviour prevented HCPs from intensifying physical activity treatment to improve their health outcomes [42, 52, 56, 65, 68]. HCPs′ concerns about the potential negative consequences of physical activity for their patients, particularly those with comorbidities or low physical fitness or ability, may discourage them from promoting it [43, 46, 48, 51, 55, 56, 63, 65]. In three studies, HCPs who felt that their physical activity advice had a positive impact on their patients′ behaviour or health outcomes were more likely to promote it [46, 57, 65].

#### 3.4.4. Social/Professional Role and Identity (Reflective Motivation)

There was a lack of consensus on who should be responsible for physical activity promotion, as well as uncertainties about the practicalities of this role [41, 46, 50, 54]. In one study, this lack of clarity led HCPs to focus more on metabolic outcomes or diet, assuming that another HCP had already discussed physical activity with the patients [46]. Another study highlighted tensions between HCPs in different roles; although some were proactive about promoting physical activity, others felt this was a patient‐ or public‐healthcare responsibility [51]. HCPs also identified the complexity of diabetes treatment within their roles as a barrier, due to the need to address multiple areas with patients, such as medications, glucose monitoring, screening and prevention of complications, managing comorbidities and complications and lifestyle education, as well as coordinating with other team members and specialists [66].

HCPs who were more physically active compared with HCPs who were not promoted physical activity more to their patients with Type 2 diabetes. They were also more confident promoting physical activity than HCPs who were not physically active [47, 64], and believed their advice had more impact on patient behaviour [46, 57]. HCPs′ personal experiences of physical activity challenges facilitated the promotion of physical activity, as they enabled HCPs to understand patients′ struggles and recognise the need to provide enhanced guidance [51].

#### 3.4.5. Skills (Physical Capability)

HCPs′ belief that they lacked the skills to promote physical activity was reported as a barrier in seven studies [40, 41, 43, 46, 54, 57, 58]. These include the ability to prescribe physical activity [57], provide physical activity counselling and use behaviour change skills and strategies in consultations, particularly with patients with comorbidities [41, 43, 46, 54]. Despite recognising the theoretical value, incorporating behaviour change skills such as motivational interviewing into patients′ appointments was challenging in practice [54] [54]. In one study, expatriate doctors in Saudi Arabia reported limited ability to provide culturally acceptable advice, especially to female patients, and difficulty applying cultural knowledge to tailor physical activity plans when patient barriers arose [40].

GPs found it challenging to communicate complex information about physical activity and Type 2 diabetes to patients despite feeling knowledgeable about the underlying physiological mechanisms [42]. They felt this barrier hindered patients′ uptake of physical activity advice, prompting the GPs to recognise the need for more training to effectively communicate information to patients and to be able to tailor this to their patient′s needs and abilities [42]. Conversely, two studies highlighted skills that facilitated more effective communication and adherence to physical activity advice, including building rapport, developing trust between the HCP and the patient, providing patient‐centred care rather than instructive advice‐giving and asking open‐ended questions [51, 54].

#### 3.4.6. Beliefs About Capabilities (Reflective Motivation)

Participants in nine studies perceived that a lack of confidence and ability was a barrier to physical activity promotion [41, 46, 54, 56, 57, 59, 64, 66, 67]. HCPs reported a perceived lack of capability to engage patients in physical activity and influence their patients′ behaviour [54, 59, 64]. GPs in one study shared that they did not feel comfortable advising their patients about the physical activity recommendations and, as a result, did not do so [57]. Lower physical activity counselling efficacy was reported by diabetes educators who included physical activity counselling in ≤ 25% of their consultations, whereas those who included it in ≥ 50% of their consultations reported higher perceived counselling efficacy [56]. Finally, due to HCPs′ unsuccessful attempts in the past to influence patients′ physical activity behaviour, they reported that prescribing medication was considered more accessible and more effective than advising patients about lifestyle behaviour change for diabetes management [66].

#### 3.4.7. Optimism (Reflective Motivation)

HCPs in four studies reported pessimistic beliefs about their patients′ motivation to be physically active, their compliance with physical activity advice and that patients were more adherent to pharmacological management of Type 2 diabetes [44, 51, 57, 68]. Consequently, these HCPs found supporting patients to be physically active challenging and, as a result, were less likely to promote it during appointments.

#### 3.4.8. Goals (Reflective Motivation)

In seven studies, HCPs viewed goal setting with patients as an effective strategy to motivate them to be physically active [42, 44, 46, 51, 52, 54]. Specific strategies used by HCPs included setting specific, measurable, achievable, realistic and timely (SMART) goals [46, 48, 54], setting goals linked to tangible outcomes such as HbA1c levels [44, 46], creating action plans at each appointment [42] and integrating physical activity goals into patients′ daily life, for example, biking to the supermarket instead of walking [51].

#### 3.4.9. Memory, Attention and Decision Processes (Psychological Capability)

In two studies [66, 68], HCPs reported difficulties prioritising physical activity promotion in patient appointments due to the complexities of diabetes treatment and the multiple aspects of care they are responsible for. One study noted that HCPs left patients to address this component of diabetes management on their own [68]. In another study, diabetes educators ranked promoting physical activity as less important than educating patients about healthy eating and taking medications [64]. Although in another study, diabetes educators reported that their patient education was dominated by ‘survival skills’, with patients being either too ill or too overwhelmed to focus on physical activity promotion [65].

#### 3.4.10. Social Influences (Social Opportunity)

Evident from the studies in this theme was that several factors related to cultural beliefs and priorities and gender and cultural expectations were barriers to physical activity promotion for HCPs [40, 41, 43–45, 48, 51, 68]. For instance, a study from Ghana reported that physical activity is considered something for wealthy people or is part of Western culture, not their own [48]. In Oman, rapid urbanisation, sedentary jobs and domestic helpers were perceived as barriers to creating a physically active culture [41]. In rural areas in South Australia, HCPs reported that social stigma and feeling embarrassed to exercise in public places were barriers to physical activity promotion [45]. Additional barriers reported in these studies were religion and gender norms, which were compounded by a lack of family support [43, 44, 48, 68]. However, cultivating a physical activity culture was reported as a facilitator to physical activity promotion, with practical approaches being the creation of walking groups [51], and creating a physical activity culture within health centres (e.g., physical activity discussions with colleagues and active meetings) [41].

The impact of HCPs′ familiarity with their patients′ social and cultural norms can be either a barrier or facilitator to physical activity promotion. In one study, despite expatriate HCPs reporting some familiarity with patients′ customs and culture, they lacked direct experience living it [40]. It was suggested that this may affect the patient′s attitude towards the physician, with them not always trusting the advice they are receiving [40]. Cultural differences, socioeconomic status and patients′ lived experiences were also highlighted as barriers to promoting physical activity. Some HCPs found it challenging to see the barriers their patients faced from their perspective, which led to conflicts with some patients.

#### 3.4.11. Emotion (Automatic Motivation)

HCPs described several negative emotions regarding physical activity promotion [42, 46, 51, 52, 57].

In some studies, participants reported feeling frustrated and irritated that their patients did not act on the physical activity advice they were given, making it difficult for them to retain the motivation to promote it [51]. Feelings of guilt were also reported, stemming from unsuccessful past efforts to change their patient′s physical activity behaviour, which led them to focus more on pharmacological management than on lifestyle change [52]. HCPs in French Guiana reported feeling isolated in physical activity promotion, as they were working in remote areas serving isolated villages [57] and dissatisfaction that their patients were unresponsive to their advice [42]. HCPs also reported feeling frustrated and overwhelmed by ‘the numerous and overlapping physical activity strategies that have been published’, making it difficult to know which one to follow [46].

#### 3.4.12. Behavioural Regulation (Psychological Capability)

In one study, physical activity was not included in the electronic health system, which was seen as a barrier to promoting physical activity, as it made it more challenging for HCPs to follow‐up, monitor, or evaluate their patients′ physical activity levels [41]. In another study, HCPs reported that smartphone health applications could facilitate patients′ tracking, monitoring and accountability for physical activity and, as such, recommended their use [62].

## 4. Discussion

This MMSR has explored and synthesised HCPs′ barriers and facilitators to physical activity promotion for adults with Type 2 diabetes using the TDF as an a priori framework [15]. The review identified 29 papers for inclusion from quantitative, qualitative and mixed‐methods studies within the published literature. Barriers were found in all TDF domains except intentions and reinforcement; the highest‐ranked domain was the environmental context and resources. The top five ranking domains, environmental context and resources, beliefs about consequences, knowledge, social/professional role and identity and skills, represented 85% of barriers and facilitators, which indicates these are key domains that should be targeted in interventions to support HCP physical activity promotion for people with Type 2 diabetes.

In line with prior research, this MMSR identified several organisation‐ and system‐level barriers to HCPs′ physical activity promotion, including limited consultation time, increasing workloads, competing clinical demands [70–72], a lack of referral pathways [73, 74] and insufficient staffing or funding [23]. HCPs also described role complexity and a lack of clarity regarding their responsibilities for promoting physical activity within routine care [71]. Collectively, these findings highlight that many barriers to promoting physical activity are not attributable to individual HCPs′ capability or motivation alone, but stem from broader sociopolitical contexts within healthcare systems [75]. Time constraints, workload pressures and unclear role expectations reflect broader structural and resourcing decisions that shape what is feasible within clinical encounters. This necessitates policy‐level action, for example, embedding physical activity promotion into diabetes care pathways as a mandated component, protected consultation time for lifestyle discussions or dedicated funding for system‐level pharmacological interventions. Future research should examine policymakers′ views and experiences to identify barriers and enablers to system‐level change that increase support for HCPs in this area of their professional practice. Such work could inform how physical activity promotion is feasibly integrated into existing care pathways and how organisational and resourcing decisions shape routine practice.

HCPs′ beliefs about their patients also influence whether physical activity is prioritised or omitted during consultations. This review demonstrates the interplay between HCPs′ perceptions of their patients′ barriers and actual patient barriers, suggesting that decisions about physical activity promotion are often shaped by anticipatory judgements rather than explicit patient needs. Although prior research has found that individuals with Type 2 diabetes report significantly more barriers to physical activity than the general population, such as lack of time [9], perceived difficulty of physical activity [76], physical discomfort [77] and lack of willpower or habit [78], these do not necessarily indicate unwillingness or inability to engage. Psychological research on stereotyping and clinical decision‐making suggests HCPs may rely on heuristics when time‐pressured, leading to systematic underestimation of patient motivation [79]. This misalignment between perceived and actual patient needs may result in physical activity being deprioritised or avoided in consultations. Addressing these beliefs will necessitate interventions beyond awareness raising, including structured reflective practice, standardised patient readiness assessments and facilitated peer supervision. Future research should explore strategies to reduce this cognitive bias and improve the equity of physical activity promotion in routine diabetes care.

HCPs also reported a lack of knowledge and skills to promote physical activity effectively, challenges often exacerbated by inadequate medical education and CPD, which is consistent with barriers reported in wider literature [23, 70, 72, 80, 81]. Evidence from an umbrella review identified inadequate or unclear guidelines as a prominent barrier across multiple systematic reviews [23]. Similarly, in this review HCPs reported that existing physical activity guidelines lack detailed information on managing Type 2 diabetes and failed to account for the multiple comorbidities and complications that many patients experience. These findings suggest that knowledge deficits are less about awareness of physical activity recommendations and more about the applicability and usability of guidance in real‐world clinical contexts. Addressing this gap will require greater attention to the content, format and dissemination of guidance, alongside embedding practical, context‐specific training within existing professional development structures to better support routine implementation.

The literature emphasises embedding physical activity education and training into healthcare systems as a priority, yet much of the available training remains predominantly pharmacologically focused [80]. Although pharmacological knowledge is important, evidence from this MMSR and existing research [72, 80] highlights the need for more comprehensive and widely accessible physical activity promotion training. This should incorporate behaviour change and communication skills to help HCPs translate knowledge into practice and build confidence in promoting physical activity to patients with Type 2 diabetes. However, improving education and training alone is insufficient [80] and capacity‐building initiatives must also address organisational and structural barriers, such as limited time, inadequate funding and an insufficient workforce. In practice, this could involve integrating physical activity promotion into mandatory diabetes competency standards, establishing interprofessional training within existing clinical settings and creating communities of practice to support skills development and shared learning. Without addressing these broader system‐level constraints, individual‐level interventions are unlikely to drive significant or sustainable change [82].

Despite barriers outweighing facilitators in this review, some HCPs identified strategies or resources that supported the promotion of physical activity. Goal setting was a key strategy for helping patients increase physical activity, with evidence linking it to reductions in HbA1c [83]. Upskilling HCPs in behaviour change strategies, such as goal setting, could enhance promotion efforts and improve patient outcomes. As commonly seen in the literature, this MMSR also identified that HCPs′ physical activity behaviour was a driver of its promotion, with physically active HCPs more likely to promote it [84, 85]. This highlights the influence that HCPs′ beliefs and behaviours can have on physical activity promotion [86] and demonstrates the need to design interventions that not only equip HCPs with the knowledge and tools to promote activity but also target their own health behaviours as a catalyst for patient‐level change. Establishing peer support structures and champion networks within healthcare settings could further enhance this effect by modelling desirable behaviours, reinforcing social norms and increasing collective accountability for promoting physical activity [87].

### 4.1. Strengths and Limitations

To the best of the researchers′ knowledge, this is the first MMSR and synthesis of barriers and facilitators experienced by HCPs in promoting physical activity for adult patients with Type 2 diabetes. There are several strengths to this review.

There were no barriers or facilitators that could not be mapped to one or more TDF domains, suggesting that the TDF provides a comprehensive account of the barriers and facilitators to physical activity promotion experienced by HCPs. Determining the importance of the TDF domains allows insight into the most significant barriers and facilitators to HCP′s physical activity promotion, which can guide intervention design, implementation and evaluation [69]. Mapping the findings to the COM‐B model provides a robust, theory‐informed methodology to then select behaviour change techniques and intervention functions to target the most influential barriers and facilitators to HCP professional behaviour change [88].

Adopting the JBI approach to MMSRs enabled the synthesis of findings from quantitative, qualitative and mixed‐methods designs, offering a novel approach to research in this domain and yielding nuanced insights that neither method could provide alone [25]. Furthermore, JBI′s evidence‐implementation approach emphasises translating research into practice through structured, theory‐informed yet flexible frameworks that guide real‐world application. This MMSR facilitates more transparent communication, making research more accessible to decision‐makers and practitioners and bridging the gap between evidence and practice [89].

Despite the strengths of this MMSR there were also limitations. The TDF is a theoretical framework that identifies and describes influences on behaviour; it is not a theory and, as such, does not propose testable relationships between elements [15]. This can make it challenging to determine the cause of a facilitator or a barrier [90]. As such, prior to the development of interventions to address HCPs′ physical activity promotion, qualitative research that uses the TDF as a framework is recommended to explore and further clarify the influences on HCPs behaviour [15, 90, 91].

The primary studies included in this MMSR did not use the TDF to assess HCPs′ professional practice; therefore, behavioural determinants were mapped to the TDF domains using a deductive approach. Although this structured, theory informed methods supports systematic comparison across studies and facilitates the identification of key influences, it also introduces potential limitations, as applying a predefined framework may constrain interpretation and emphasise constructs that fit within existing domains. To mitigate this, coding incorporated both deductive mapping to TDF domains and inductive consideration of study findings, with a second reviewer independently reviewing coded data to enhance rigour.

Nonetheless, some TDF domains may have been underrepresented as the primary studies did not include questions that captured the relevant domains and their component constructs. For example, previous studies have reported that HCPs experience clinical inertia [92]. This would relate to the emotion domain; however, this was not identified as an important domain in this MMSR. Additionally, the focus of some studies on specific areas such as knowledge [67] or resources [41] may have disproportionately contributed to the frequency of those domains, potentially overlooking barriers or facilitators in less‐represented TDF domains. For future studies, a TDF‐based questionnaire or interview schedule may facilitate a broader understanding of barriers and facilitators.

It is important to note that the paucity of facilitators found in this MMSR may not be representative of HCPs′ actual experience, as not all included studies were designed to explore barriers and facilitators explicitly. Nonetheless, the lack of facilitators highlights a critical challenge some HCPs face when promoting physical activity. Global guidelines state that HCPs should advise, encourage, support and counsel diabetic patients on physical activity as part of ongoing diabetes education and self‐management [11]. However, as demonstrated in this review and the wider literature, they often report multiple barriers and fewer facilitators to this in practice [70, 72].

## 5. Conclusion

This MMSR provides a theory‐informed synthesis of the barriers and facilitators influencing HCPs′ promotion of physical activity for people with Type 2 diabetes. Application of the TDF [15] and COM‐B model [19] supports a more explanatory understanding of why physical activity promotion remains challenging in routine care, highlighting the interaction between individual, organisational and system‐level influences on clinical practice. The findings demonstrate that barriers are pervasive, multifaceted and frequently intersecting, whereas evidence‐based facilitators to support HCPs are limited and inconsistently reported [72].

Collectively, these findings suggest that efforts to improve physical activity promotion within routine diabetes care are unlikely to be effective if focused solely on individual knowledge or motivation. Rather, progress will require developing and evaluating theory‐based strategies that address behavioural determinants across multiple levels, including organisational processes and service delivery contexts, to support sustainable change in everyday clinical practice.

## Funding

No funding was received for this manuscript.

## Conflicts of Interest

The authors declare no conflicts of interest.

## Supporting Information

Additional supporting information can be found online in the Supporting Information section.

## Supporting information


**Supporting Information 1** Appendix S1: Search strategies for the MMSR.

**Supporting Information 2** Appendix S2: Quality assessment of the qualitative, Quantitative and mixed‐methods studies.

**Supporting Information 3** Appendix S3: Themes and subthemes (with number of studies) identified as barriers or facilitators in TDF domains.

**Supporting Information 4** Appendix S4: Overview of the findings for the TDF domains (and COM‐B model) with exemplar quotes.

**Supporting Information 5** PRISMA statement 2020.

## Data Availability

The data that support the findings of this study are openly available in BCU Open Access at https://www.open-access.bcu.ac.uk/id/eprint/16095.
