# Supplementary material for: Barriers and Facilitators to the Delivery of Physical Activity Promotion by Healthcare Professionals for Adults With Type 2 Diabetes: A Mixed‐Methods Systematic Review Using the Theoretical Domains Framework
Source: J Diabetes Res. 2026 Mar 23;2026:4048417. doi: 10.1155/jdr/4048417 (PMC13140809; doi:10.1155/jdr/4048417)
Supplement: Supplementary file 1 — Supporting Information 1 Appendix S1: Search strategies for the MMSR. [file JDR-2026-4048417-s002.pdf]

Appendix 1

Search Strategies for the MMSR

Search strategy for MEDLINE and PubMed

#1 EXP “Health personnel” [Mesh]
#2 EXP “Health occupations” [Mesh]
#3 OR/ 1-2
#4 EXP “Diabetes mellitus, Type 2” [Mesh]
#5 type 2 diabetes or type 2 diabetes mellitus or t2dm [Keywords]
#6 OR/4-5
#7 EXP “Exercise” [Mesh]
#8 EXP “Sports” [Mesh]
#9 “Sedentary behavio#r*” [Mesh]
#10 EXP “Exercise therapy” [Mesh]
#11 EXP “Physical fitness” [Mesh]
#12 Lifestyle [Keyword]
#13 OR/ 7-12
#14 “Intervention*” [Keyword]
#15 EXP “Early medical intervention” [Mesh]
#16 EXP “Internet based intervention” [Mesh]
#17 EXP “Health promotion” [Mesh]
#18 EXP “Patient care management” [Mesh]
#19 EXP “Counsel#ing” [Mesh] or counsel* [keyword]
#20 EXP “Program evaluation” [Mesh]
#21 EXP “Health education” [Mesh]
#22 EXP “Delivery of health care” [Mesh]
#23 OR/14-22
#24 EXP “Behavio#r and behavio#r mechanisms” [Mesh]
#25 EXP “Education” [Mesh]
#26 EXP “Organization and administration” [Mesh]
#27 EXP “Health communication” [Mesh]
#28 EXP “Psychological phenomenon” [Mesh]
#29 OR/ 24 -28
#3 AND #6 AND #13 AND #23 AND #29

Search strategy for CINHAL

#1 EXP “Health personnel” [Mesh]
#2 EXP “Health occupations” [Mesh]
#3 OR/ 1-2
#4 EXP “Diabetes mellitus, Type 2” [Mesh]
#5 type 2 diabetes or type 2 diabetes mellitus or t2dm [Keyword]

#6 OR/4-5
#7 EXP “Exercise” [Mesh]
#8 EXP “Physical Activity’’ [Mesh]
#9 EXP “Sports” [Mesh]
#10 “Life style sedentary” [Mesh]
#11 Lifestyle [Keyword]
#12 EXP “Therapeutic exercise” [Mesh]
#13 EXP “Physical fitness” [Mesh]
#14 OR/ 7-13
#15 “Intervention*” [Keyword]
#16 EXP “Early medical intervention” [Mesh]
#17 EXP “Internet based intervention” [Mesh]
#18 EXP “Intervention trials’’ [Mesh]
#19 EXP “Nursing interventions” [Mesh]
#20 EXP “Health promotion” [Mesh]
#21 EXP “Patient care management” [Mesh]
#22 EXP “Counsel#ing” [Mesh] or counsel* [keyword]
#23 EXP “Program evaluation” [Mesh]
#24 EXP “Health education” [Mesh]
#25 EXP “Health care delivery” [Mesh]
#26 OR/15-25
#27 EXP “Behavio#r and behavio#r mechanisms” [Mesh]
#28 EXP “Education” [Mesh]
#29 EXP “Health facility administration and management” [Mesh]
#30 EXP “Health communication” [Mesh]
#31 EXP “Psychological phenomenon” [Mesh]
#32 OR/ 27 -31
#3 AND #6 AND #14 AND #26 AND #32

Search strategy for PsycINFO:

#1 EXP “Health personnel”
#2 EXP “Medical personnel”
#3 OR/ 1-2
#4 EXP “Diabetes mellitus, Type 2”
#5 type 2 diabetes or type 2 diabetes mellitus or t2dm [Keyword]
#6 OR/4-5
#7 EXP “Exercise”
#8 EXP “Sports”
#9 EXP “Sedentary behaviour”
#10 Lifestyle [Keyword]
#11 EXP “Exercise therapy”
#12 EXP “Physical fitness”
#13 OR/ 7-12
#14 “Intervention*” [Keyword]
#15 EXP “Internet based intervention”

#16 EXP “Health promotion
#17 EXP “Patient care management” [Mesh]
#18 EXP “Counsel#ing”
#19 Counsel* [keyword]
#20 EXP “Program evaluation”
#21 EXP “Health education”
#22 EXP “Delivery of healthcare”
#23 OR/ 13-22
#24 EXP “Behavio#r and behavio#r mechanisms”
#25 EXP “Education”
#26 EXP “Organi#ation and administration”
#27 EXP “Health communication”
#28 EXP “Psychological phenomenon”
#29 OR/ 24 -28
#3 AND #6 AND #13 AND #23 AND #29

Search strategy for Web of Science

#1 Healthcare professionals or health-care professionals or health professionals
#2 Health Personnel
#3 Health care workers
#4 Nurs* or nurse practitioner
#5 General practitioner or doctor or physician or family practice
#6 Community health workers or allied health or dieticians or physiotherapy* or pharmacist
#7 Primary care or public health or public health practice
#8 OR/1-7
#9 Diabetes Mellitus, type 2
#10 Type 2 diabetes or diabetes type 2 or type 2 diabetes mellitus
11 metabolic health or glycaemic control or insulin resistance or HBA1c
#12 T2D or T2DM or NIDDM
#13 OR/9-12
#14 Physical activity
#15 Exercise
#16 Physical Fitness
#17 Sports
#18 Lifestyle advice
#19 Lifestyle or life style
#20 Pedometer
#21 Exercise therapy
#22 Sedentary behaviour
#23 OR/ 14-22
#24 Intervention or program or trial or study or prevention
#25 Experiences of health care professionals
#26 Behavio$r change or health behavio$r or behavio$r therapy
#27 Patient-provider education or health education or patient education as topic or counselling or

counsel*
#28 Prescription
#29 Health communication or health promotion
#30 Evaluation or process evaluation
#31 Patient care planning
#32 Patient compliance
#33 OR/24-32
#8 AND #13 AND #23 AND 33
