# Supplementary material for: Barriers and Facilitators to the Delivery of Physical Activity Promotion by Healthcare Professionals for Adults With Type 2 Diabetes: A Mixed‐Methods Systematic Review Using the Theoretical Domains Framework
Source: J Diabetes Res. 2026 Mar 23;2026:4048417. doi: 10.1155/jdr/4048417 (PMC13140809; doi:10.1155/jdr/4048417)
Supplement: Supplementary file 2 — Supporting Information 2 Appendix S2: Quality assessment of the qualitative, Quantitative and mixed‐methods studies. [file JDR-2026-4048417-s003.pdf]

## Appendix 2

## Quality Assessment of the Qualitative, Quantitative and Mixed-Methods Studies

| Qualitative Studies | | | | | | | | | | | Score | % | Quality |
|---|---|---|---|---|---|---|---|---|---|---|---|---|---|
| **Study** | **Q1** | **Q2** | **Q3** | **Q4** | **Q5** | **Q6** | **Q7** | **Q8** | **Q9** | **Q10** | | | |
| Abouammoh et al. (2016) | U | U | Y | Y | Y | N | Y | Y | Y | Y | 7/10 | 70 | High |
| Alghafri et al. 2017) | U | Y | Y | Y | Y | N | Y | Y | Y | Y | 8/10 | 80 | High |
| Avery (2014) | U | Y | Y | Y | Y | N | N | Y | Y | Y | 7/10 | 70 | High |
| Berry et al. (2012) | U | Y | Y | Y | Y | N | N | Y | Y | Y | 7/10 | 70 | High |
| Carbone et al. (2007) | U | Y | Y | Y | Y | N | Y | Y | Y | Y | 8/10 | 80 | High |
| Jones et al. (2014) | U | Y | Y | Y | Y | N | N | Y | Y | Y | 7/10 | 70 | High |
| Larme and Pugh (1998) | U | Y | Y | N | Y | N | N | N | N | Y | 4/10 | 40 | Moderate |
| Matthews et al. (2014) | U | Y | Y | Y | Y | N | N | Y | Y | Y | 7/10 | 70 | High |
| Miller and Beech (2009) | U | Y | Y | N | Y | N | N | Y | Y | Y | 6/10 | 60 | Moderate |
| Mogre et al. (2019) | U | Y | Y | Y | Y | N | Y | Y | Y | Y | 8/10 | 80 | High |
| Paiva et al. (2019) | U | Y | Y | Y | Y | N | Y | Y | Y | Y | 8/10 | 80 | High |
| Raaijmakers et al. (2013) | U | Y | Y | Y | Y | N | N | Y | Y | Y | 7/10 | 70 | High |
| Stuij (2018) | U | Y | Y | Y | Y | N | Y | Y | Y | Y | 8/10 | 80 | High |
| Svenningsson et al. (2011) | U | Y | Y | Y | Y | N | Y | Y | Y | Y | 8/10 | 80 | High |
| Torres et al. (2010) | U | Y | Y | Y | Y | N | N | Y | Y | Y | 7/10 | 70 | High |
| Zimmerman et al. (2018) | U | Y | Y | Y | Y | N | N | Y | Y | Y | 7/10 | 70 | High |

| Cross-Sectional Studies (Quantitative Studies) | | | | | | | | | Score | % | Quality |
|---|---|---|---|---|---|---|---|---|---|---|---|
| **Study** | **Q1** | **Q2** | **Q3** | **Q4** | **Q5** | **Q6** | **Q7** | **Q8** | | | |
| Armstrong-Schultz et al. (2001) | Y | Y | Y | Y | N | N | Y | Y | 6/8 | 75 | High |
| Dillman et al. (2010) | U | Y | U | Y | N | N | U | Y | 3/8 | 38 | Moderate |
| Doehring et al. (2016) | N | Y | U | Y | Y | N | U | Y | 4/8 | 50 | Moderate |
| Dranebois et al. (2019) | Y | Y | U | Y | N | N | U | Y | 4/8 | 50 | Moderate |
| George et al. (2006) | Y | Y | N/A | Y | N | N | Y | Y | 5/7 | 71 | High |
| Gross et al. (2007) | Y | Y | N | Y | Y | Y | U | Y | 6/8 | 75 | High |
| Hixenbaugh and Winkley (2001) | Y | Y | U | Y | N | N | Y | Y | 5/8 | 63 | Moderate |
| Hnatiuk et al. (2012) | Y | Y | U | U | N | N | U | Y | 3/8 | 38 | Moderate |
| Karduck and Chapman-Novokofski (2018) | Y | Y | U | U | N | N | U | Y | 3/8 | 38 | Moderate |
| Khairnar et al. (2018) | N | Y | Y | Y | N | N | Y | Y | 5/8 | 63 | Moderate |
| Lanhers et al. (2015) | Y | N | Y | U | N | N | U | Y | 3/8 | 38 | Moderate |
| Larme and Pugh (1998) | N | Y | U | U | N | N | U | Y | 2/8 | 25 | Low |
| Powell et al. (2016) | Y | Y | U | U | N | N | U | Y | 3/8 | 38 | Moderate |
| Ruby et al. (1993) | Y | Y | Y | Y | N | N | U | Y | 5/8 | 63 | Moderate |

Note. Y = Yes, N = No, U – Unclear
