# Supplementary material for: Barriers and Facilitators to the Delivery of Physical Activity Promotion by Healthcare Professionals for Adults With Type 2 Diabetes: A Mixed‐Methods Systematic Review Using the Theoretical Domains Framework
Source: J Diabetes Res. 2026 Mar 23;2026:4048417. doi: 10.1155/jdr/4048417 (PMC13140809; doi:10.1155/jdr/4048417)
Supplement: Supplementary file 3 — Supporting Information 3 Appendix S3: Themes and subthemes (with number of studies) identified as barriers or facilitators in TDF domains. [file JDR-2026-4048417-s004.pdf]

Appendix 3

Exemplar Quotes for TDF Domain (COM-B component) Barriers and Facilitators

TDF Domain (COM-B)	Theme (n studies)	Type	Exemplar Quotes
Knowledge (Psychological Capability)	General knowledge about physical activity (13)	Barrier (23)<br>Facilitator (3)	Twenty-nine per cent of diabetes educators reported that their own lack of ability or knowledge was a barrier to physical activity promotion in appointments with type 2 diabetic patients (Dillman et al., 2010)<br><br>"Health Canada says, 'participate in moderate aerobic-intensity-level activity three times a week and do resistance exercise three times a week'...I basically look at them at that point and say, 'Okay, does that make sense to you? Because it doesn't make sense to me either, right, because when you read that thing, you're like, Whaaatt? Like it's not clear as to what you do" (Berry et al., 2012)<br><br>Limited knowledge of physical activity effects on diabetes control was a barrier to physical activity counselling for the healthcare professionals (Powell et al., 2016)
	Knowledge to support patients with comorbidities or complications (5)	Barrier (5)	A challenging barrier to physical activity counselling for diabetes educators was assuring safe physical activity plans for patients with comorbidities (Powell et al., 2016)<br><br>"I mean, a lot of them can be in wheelchairs or on walking sticks, and physical activity would not be possible or a priority with them, so that would probably be the main reason [why physical activity is not discussed]" (Matthews et al., 2014)

TDF Domain (COM-B)	Theme (n studies)	Type	Exemplar Quotes
			"Am not sure about my knowledge and skills to support physical activity in patients with diabetes who may have multiple comorbidities and require structured physical activity advice, not just a general statement" (Alghafri et al., 2017)
	Impact of inadequate training and education on knowledge (5)	Barrier (6)	"It should be well-structured physical activity consultations. I think no one is well trained in this field" (Alghafri et al., 2017)<br><br>A lack of physical activity and exercise training for diabetes management was observed: 40% had received no formal training, while another 40% had only received one form of training (Dillman et al., 2010)<br><br>Providers complained that they had received insufficient training in medical school and in their residencies to promote behavioral change (Larme et al., 1998)
	Knowledge of the social and environmental context (2)	Facilitator (3)	One strategy put forward by a male IMG was to ask male patients to walk to a mosque that was further away than the mosque they usually attend which would indicate that the IMGs awareness of local people's religious needs (Abouammoh et al., 2016)<br><br>Community mapping for physical activity facilities (places and volunteering buddies) to inform healthcare providers is a good idea to improve PA referrals (Alghafri et al., 2017)

TDF Domain (COM-B)	Theme (n studies)	Type	Exemplar Quotes
Skills (Psychological Capability)	Behaviour change skills required to promote physical activity (7)	Barrier (10)	Thirty-four percent of physicians did not feel comfortable applying the physical activity recommendations with their type 2 diabetic patients and, as such, did not do so, reporting a lack of appropriate skills in physical activity prescription (Dranebois et al., 2019)<br><br>Despite health professionals having sufficient knowledge to provide general physical activity information, they recognised their limited skills in delivering effective behaviour change consultations (Matthews et al., 2014)<br><br>"I know that physical activity consultations linked to behaviour change is more effective, but we don't know how to do it" (Alghafri et al., 2017)
	Communication skills (3)	Barrier (1) Facilitator (5)	GPs reported feeling knowledgeable about the underlying physiological mechanisms of Type 2 diabetes, however they emphasised that it was sometimes difficult to communicate this complex information to patients. Furthermore they expressed dissatisfaction that many of their patients do not act upon the advice they provide about increasing their PA/exercise levels. As such they felt that a different approach was required to effectively communicate information about diabetes to patients, including the benefits of leading a physically active lifestyle that would be more flexible to patients' personal situations (Avery, 2014)<br><br>"My number one aim is just to build rapport. I always feel that if people trust you and inherently like who you are, they are probably going to be more adherent because they enjoy coming" (Zimmermann et al., 2018)

TDF Domain (COM-B)	Theme (n studies)	Type	Exemplar Quotes
Social/Professional Role and Identity (Reflective Motivation)	HCPs perception of their roles and responsibilities (6)	Barrier (18)	"Getting people to be active is a public healthcare responsibility. My responsibility is to offer these people, when they have developed diabetes, their care as good as possible … not to change their [patients] behaviour. Even if you do your absolute best, it won't happen" (Stuij, 2018)<br><br>"You just have two categories of people: those who sit behind the wheel, who want to have control over their life, have their own responsibility and take it, and there are people who sit in the back of the bus and let themselves be driven, who let it happen. And if those people don't undergo a change [in attitude], they have a long way to go before they get in at the front of the bus, sit there. Then you have such a different way to go before you even talk about PA" (Stuij, 2018)
	HCPs physical activity behaviour (5)	Facilitator (7)	All the physicians who practised regular physical activity thought that their recommendations had an impact versus 70% of physicians who did not practice regular physical activity (Dranebois et al., 2019)<br><br>Diabetes educators engaging in regular physical activity (at least over the past 6 months) perceived themselves as more confident counseling on physical activity compared with those who reported not engaging in regular physical activity over the past 6 months (p = .002) (Powell et al., 2016)
Beliefs about Capabilities (Reflective Motivation)	HCPs beliefs about their ability and confidence to promote	Barrier (11)	In general, HCPs reported lacking in confidence when tackling lifestyle issues [physical activity] with their patients, primarily due to frustration resulting from numerous unsuccessful attempts in the past (Avery, 2014)

TDF Domain (COM-B)	Theme (n studies)	Type	Exemplar Quotes
	physical activity (9)		Diabetes educators lacked confidence in both their ability to prescribe physical activity and exercise and their ability to make appropriate physical activity and exercise-related referrals, with both averages being below the midpoint of the scale (i.e., 50%) (Dillman et al., 2010)
Optimism (Reflective Motivation)	Pessimistic beliefs about the impact of physical activity advice on patient behaviour (4)	Barrier (10)	The lack of motivation from the patient made the practitioner pessimistic about being able to manage this type of patients. Another respondent noted: Patients find it difficult to curb their appetite for good tasting bad foods and prefer to watch TV than exercise (Khairnar et al., 2018)<br><br>"That's what you get, especially people with type 2 diabetes, nine out of ten revert to the same habits. So, I'm pessimistic about it. But in this case, it's realistic. (…) I do my best for the people I see, but, in the end, I won't create a solution with my advice" (Stuij, 2018)
Beliefs about Consequences (Reflective Motivation)	Patients interest and motivation for physical activity (12)	Barrier (20)	Diabetes educators perceived patients to be less receptive to physical activity and exercise for diabetes self-management than themselves (4.5 ± versus 3.2 ±0.7) (Dillman et al., 2010)<br><br>"Of all the diseases out there, diabetes is the one they are going to do kicking and screaming into making lifestyle changes…When I say exercise or even activity, the walls are up" (Berry et al., 2012)<br><br>One hundred per cent of diabetes educators perceived that exercise is not a priority for type 2 diabetic patients (Armstrong-Schultz et al., 2001)

TDF Domain (COM-B)	Theme (n studies)	Type	Exemplar Quotes
	Patients adherence to physical activity advice (5)	Barrier (7)	The PCPs believed that <50% of their patients were adherent to regular moderate exercise (Khairnar et al., 2018)<br><br>GPs expressed dissatisfaction that many of their patients do not act upon the advice they provide about increasing their PA/exercise levels (Avery, 2014)
	Patient complications and comorbidities (8)	Barrier (25)	General practitioners reported that a patient's low fitness level (3.06 ± 0.3) was a barrier that would stop them prescribing regular physical activity to their type 2 diabetes patients (Lanhers et al., 2015)<br><br>Comorbidities such as arthritis, obesity, and cardiovascular disease were also mentioned as barriers to being active. Overall, there was a general recognition that changing physical activity behaviour is very difficult and that participants "can't be rushed" (Berry et al., 2012)<br><br>Ninety-nine per cent of diabetes educators perceived that the choices of activities for patients with type 2 diabetes are minimal because of physical limitations (Armstrong-Shultz et al., 2001)<br><br>"I mean a lot of them can be in wheelchairs or on walking sticks and physical activity would not be possible or a priority with them. So that would probably be the main reason [why physical activity is not discussed]" (Matthews et al., 2014)
	Belief in the impact of	Facilitator (7)	To be convinced of the interest of prescription was seen as a very important facilitator by 28.8% of participants, an important facilitator by 5.5%, an

TDF Domain (COM-B)	Theme (n studies)	Type	Exemplar Quotes
	physical activity on patient outcomes (3)		average facilitator by 8.2%, and a low facilitator by 11% (Dranebois et al., 2019)<br><br>83% of registered nurse-diabetes educators physical activity had overall benefits for diabetes management, which was a reason why they would include it in other education programs (Ruby et al., 1993)
Goals (Reflective Motivation)	Goal setting (7)	Facilitator (11)	"It's usually to address specific needs, clinical needs of the patient. So it might be somebody whose HbA1C is slight higher than we'd like. So then the benefits of increasing their physical activity might get them to their desired target" (Matthews et al., 2014)<br><br>"I think with exercise… give them a very specific timetable for what I expect them to have done by the next appointment. Because… if you just say 'I'd like you to start exercising, do some swimming'? [No good]. You need to say 'How about you do 3 sessions of swimming?'" (Avery, 2014)<br><br>"We try to tell them to make time; it starts slowly. For exercising you can start with 5 min(s) and progress from there" (Mogre et al., 2019)
Memory, Attention and Decision Processes (Psychological Capability)	Competing demands and prioritisation of physical activity (4)	Barrier (7)	"We are time pressured in our interaction with patients so we can't really cover all aspects of diabetes care with them in one visit, never mind the aspects of wider care. So it's almost a focus thing, focussing it all on blood pressure, or focussing it on foot care or something like that" (Matthews et al., 2014)

TDF Domain (COM-B)	Theme (n studies)	Type	Exemplar Quotes
			When examining the importance placed on the 4 DSME/S content areas, diabetes educators ranked healthy eating (38.3%) and taking medications (28%) as the 2 most important content areas to address ahead of physical activity (19.7%) and blood glucose monitoring (14%) (Powell et al., 2016)<br><br>Diabetes treatment is also harder for providers because it is more complex and requires close coordination with patients and specialists. There are more components to diabetes treatment (medications, glucose monitoring, education about diet and exercise, screening for and prevention of complications) than for other diseases, and diabetes has more complications and comorbidities because the biochemical changes in diabetes affect all organs in the body. The different therapies and numerous specialists required for the complications and comorbidities of diabetes are also difficult for providers to coordinate (Larme et al., 1998)
Environmental Context and Resources (Physical Opportunity)	HCPs lack of time (12)	Barrier (31)	Finally, the educators noted that for many clients making changes in diet or exercise behaviour simply wasn't a priority and, with competing demands on time, lifestyle changes fall off the list (Berry et al., 2012)
	Perceptions of patients lack of time (6)	Barrier (6)	"Our main problem is with the females, whom, they don't have the time, they don't have the place to do it, and they have many social commitments" (Alghafri et al., 2017)
	HCPs access to resources (17)	Barrier (29) Facilitator (10)	The majority of the respondents reported not to be aware of lifestyle programs and prevention initiatives that they could refer their diabetes patients to. One respondent reported "not to have a list of local exercise facilities" (Raaijmakers et al., 2013)

TDF Domain (COM-B)	Theme (n studies)	Type	Exemplar Quotes
			Forty-six per cent of RN, CDEs [registered nurse, certified diabetes educators] indicated that lack of resources (time, money, facilities, equipment, personnel, and physician and institutional support) was the most important reason why implementing a comprehensive exercise teaching program specifically for elderly clients with NIDDM [non-insulin dependent diabetes mellitus] was difficult (Ruby et al., 1993)<br><br>The term "community mapping" was used by a senior manager who thought health workers should be aware of physical activity facilities within the geographical catchment areas of primary health care centres, in order to facilitate physical activity referrals when advised (Alghafri et al., 2017)
	Perception of patients access to resources (7)	Barrier (12)	Ninety-one per cent of diabetes educators perceived that their type 2 diabetic patient doesn't have any place to exercise (Armstrong-Shultz et al., 2001)<br><br>Some health professionals indicated that exercise options are limited for rural dwellers and that a lack of diversity in options for exercise contributed to difficulty in managing type 2 diabetes: "We didn't have a swimming pool dedicated to rehab and aqua types of sports, where people, you know with joint problems or really overweight or obese people could perhaps get in the water and do some kind of exercise" (Jones et al., 2014)<br><br>All participants perceived a lack of PA facilities, particularly safe walking areas (Alghafri et al., 2017)

TDF Domain (COM-B)	Theme (n studies)	Type	Exemplar Quotes
	Financial challenges (8)	Barrier (11)	No reimbursement for physical activity counselling was reported as a barrier to physical activity counselling (Powell et al., 2016)<br><br>"But then [when the insurance fee ends after three months] people tell me, 'I quit, because I didn't get it paid anymore'. Well, yeah, you can walk outside, guys. That's what you get, especially people with type 2 diabetes, nine out of ten revert to the same habits. So, I'm pessimistic about it" (Stuij, 2018)<br><br>"Our physical activity budget, we get within the Health Board, is about 3/4million [GBP], so we put about GBP 750 000 into the physical activity, the core budget ... So that's less than a pound per person spending on physical activity within the [Health] Board. The majority of that funding will go to our exercise referral scheme for the salaries of our [physical activity] advisors" (Matthews et al., 2014)
	Organisational support and priorities (7)	Barrier (10)	Limited physician support and/or guidance for physical activity was reported as a barrier to physical activity counselling (Powell et al., 2016)<br><br>"Physical activities (are) not medicalised and hence there are no standard follow up, monitoring or evaluative tools for it in primary care" (Alghafri et al., 2017)<br><br>"We do try and get it [physical activity] in planning frameworks so that there is a responsibility for the areas [Health Boards] to do something about

TDF Domain (COM-B)	Theme (n studies)	Type	Exemplar Quotes
			physical activity. But it's patchy [across the Health Boards]" (Matthews et al., 2014)
Social Influences (Social Opportunity)	Social and cultural norms (8)	Barrier (15)<br>Facilitator (3)	Culturally, others perceive that exercise is for the rich or it is a western culture and hence will not participate in it if they think they are poor: "The practice of exercising in the Northern culture is low or non-existent if I should put it bluntly. So if someone sees you exercising (e.g. jogging) it appears strange" (Mogre et al., 2019)<br><br>"In our culture (we don't view physical activity as important), taking medicine is enough, no need for physical activity" (Alghafri et al., 2017)<br><br>"You have a woman who needs to get out to get some exercise to be healthy, but it's against Muslim...they can't go out on their own, they don't necessarily have family support, so there's... so there's cultural issues" (Berry et al., 2012)<br><br>Elena (practice nurse) started a weekly walking group herself, because she wanted to offer her patients something concrete and accessible instead of 'only telling them to be more active'. Marjolein (practice nurse) also started such a group. Both spent some of their spare time on the organisation. They were enthusiastic about their group, and felt it offered something important for some of their patients, both because of the physical activity and the social aspects (Stuij, 2018)

TDF Domain (COM-B)	Theme (n studies)	Type	Exemplar Quotes
	Awareness and understanding of social and cultural differences (2)	Barrier (8)	"If the doctor thinks with her mentality, culture and habits, she will never understand that it is socially unacceptable in Saudi Arabia for a woman over 40 years of age to visit the gymnasium" (Abouammoh et al., 2016)<br><br>"Maybe the expatriate doctor is familiar with the customs and habits but cannot link her information with providing advice to patients, it is just not in her mind because she does not live that culture, she just knows it" (Abouammoh et al., 2016)
Emotion (Automatic Motivation)	Feeling negative about physical activity promotion (5)	Barrier (9)	Fifty two percent of the physicians reported feeling isolated regarding prescribing physical activity for patients with type 2 diabetes (Dranebois et al., 2019)<br><br>GPs expressed dissatisfaction that many of their patients do not act upon the advice they provide about increasing their PA/exercise levels. As such they felt that a different approach was required to effectively communicate information about diabetes to patients, including the benefits of leading a physically active lifestyle that would be more flexible to patients' personal situations (Avery, 2014)<br><br>"We've got a national physical activity strategy and following that we've now got a kind of national cycling action plan and we're now developing a walking strategy. Why do we need a walking strategy? We've got a perfectly good physical activity strategy that references walking" (Matthews et al., 2014)

TDF Domain (COM-B)	Theme (n studies)	Type	Exemplar Quotes
Behavioural Regulation (Psychological Capability)	Tracking, monitoring and evaluation (2)	Barrier (3) Facilitator (4)	"Physical activity is not considered in the primary health information system 'ALSHIFA' which makes it difficult to prescribe, follow-up or evaluate" (Alghafri et al., 2017)<br><br>Fifty-eight per cent of clinicians recommended smartphone apps to their clients as they tracked physical activity better than traditional methods (Karduck & Chapman-Novkofski, 2018)
