# Supplementary material for: Barriers and Facilitators to the Delivery of Physical Activity Promotion by Healthcare Professionals for Adults With Type 2 Diabetes: A Mixed‐Methods Systematic Review Using the Theoretical Domains Framework
Source: J Diabetes Res. 2026 Mar 23;2026:4048417. doi: 10.1155/jdr/4048417 (PMC13140809; doi:10.1155/jdr/4048417)
Supplement: Supplementary file 4 — Supporting Information 4 Appendix S4: Overview of the findings for the TDF domains (and COM‐B model) with exemplar quotes. [file JDR-2026-4048417-s005.pdf]

Appendix 4

Themes and Subthemes (with number of studies) Identified as Barriers or Facilitators in TDF Domains

TDF Domain (COM-B)	Theme (Number of Studies)	Subtheme (Number of Studies)	Barrier/Facilitator/ Both
**Knowledge (psychological capability)**	Knowledge about physical activity (13 studies)	General knowledge about physical activity (13 studies)	Both
		Lack of knowledge to support patients with comorbidities or complications (5 studies)	Barrier
	Knowledge of the social and environmental context (2 studies)	None	Facilitator
	The impact of inadequate training and education on knowledge (5 studies)	None	Barrier
**Skills (physical capability)**	Behaviour change skills required to promote physical activity (7 studies)	None	Barrier
	Communication skills (3 studies)	None	Both
**Social/Professional Role and Identity (reflective motivation)**	HCPs' perception of their roles and responsibilities for physical activity promotion (6 studies)	None	Barrier
	HCPs' physical activity behaviour (5 studies)	None	Facilitator
**Beliefs about Capabilities (reflective motivation)**	HCPs' beliefs about their ability and confidence to promote physical activity (9 studies)	None	Barrier

**Optimism (reflective motivation)**	Pessimistic beliefs about the impact of physical activity advice on patient behaviour (4 studies)	None	Barrier
**Beliefs about Consequences (reflective motivation)**	Beliefs about patients (15 studies)	Patients interest and motivation for physical activity (12 studies)	Barrier
		Patients' adherence to physical activity advice (5 studies)	Barrier
		Patient comorbidities and complications (8 studies)	Barrier
	Belief in the impact of physical activity advise on patient outcomes (3 studies)	None	Facilitator
**Goals (reflective motivation)**	Goal setting (7 studies)	None	Facilitator
**Memory, Attention and Decision Processes (psychological capability)**	Competing demands and prioritisation of physical activity (4 studies)	None	Barrier
**Environmental Context and Resources (physical opportunity)**	Lack of time (17 studies)	HCPs time (12 studies)	Barrier
		Perceptions of patients lack of time (6 studies)	Barrier
	Access to resources (18 studies)	HCPs access to resources (17 studies)	Both
		Perceptions of patients access to resources (7 studies)	Both

	Financial challenges<br>(8 studies)	None	Barrier
	Organisational support and priorities<br>(7 studies)	None	Barrier
**Social Influences (social opportunity)**	Social and cultural norms<br>(8 studies)	None	Both
	Awareness and understanding of social and cultural differences<br>(2 studies)	None	Barrier
**Emotion (automatic motivation)**	Feeling negative about physical activity promotion<br>(5 studies)	None	Barrier
**Behavioural regulation (psychological capability)**	Tracking, monitoring and evaluation<br>(2 studies)	None	Both
